# Retrospective Analysis on the Impact of Triptorelin on Final Height of Girls with Precocious and Early Puberty: A Single-Center, Long-Term Study

**DOI:** 10.3390/children12070818

**Published:** 2025-06-21

**Authors:** Georgia Sotiriou, Anastasios Serbis, Assimina Galli-Tsinopoulou, Athanasios Christoforidis

**Affiliations:** 1 Program of Postgraduate Studies “Adolescent Medicine and Adolescent Health Care”, School of Medicine, Faculty of Health Sciences, Aristotle University of Thessaloniki, 54124 Thessaloniki, Greece; 2 First Department of Pediatrics, School of Medicine, Faculty of Health Sciences, Aristotle University of Thessaloniki, Hippokratio General Hospital, 54642 Thessaloniki, Greece; 3 Department of Pediatrics, School of Medicine, University of Ioannina, 45110 Ioannina, Greece; 4 Second Department of Pediatrics, School of Medicine, Faculty of Health Sciences, Aristotle University of Thessaloniki, AHEPA General Hospital, 54124 Thessaloniki, Greece

**Keywords:** precocious puberty, early puberty, triptorelin, GnRH analogues, final height

## Abstract

**Background/Objectives:** To evaluate the effect of triptorelin on final height of girls with precocious or early puberty, compared to the untreated group, and to investigate factors that contribute to its maximum effectiveness in terms of final height. **Methods:** We collected for the last two decades the data of patients evaluated in our Pediatric Endocrinology Clinic for precocious (PP) (thelarche before 8 years of age) or early puberty (EP) (thelarche before 9 years of age) during the last two decades. Our final set included 178 girls (85 with precocious and 93 with early puberty, of whom 85 received triptorelin). Final heights, measured and documented by health professionals, and the exact date of menarche were collected after telephone communication. Logistic regression analysis assessed the effect of various parameters on the response to treatment. **Results:** Τhe difference in mean standard deviation (ΔSDS) of final and midparental height did not show significant difference between treated and untreated girls (ΔHeight SDS (Final − Midparental): −0.20 ± 0.89 vs. −0.28 ± 0.83, *p* = 0.243). The results were similar when we compared the EP (−0.22 ± 0.71 vs. −0.17 ± 0.83, *p* = 0.778) and PP (−0.19 ± 1.04 vs. −0.39 ± 0.83, *p* = 0.315) subgroups. Menarche occurred earlier in the PP group compared to the EP group (10.68 ± 1.22 vs. 11.12 ± 0.90 years, *p* = 0.005) and in the untreated compared to the treated group (10.31 ± 0.91 vs. 11.57 ± 0.06 years, *p* < 0.001 for EP, 11.53 ± 0.90 vs. 9.86 ± 0.86 years, *p* < 0.001 for PP). Predictors of final height were height at diagnosis (positively correlated), midparental height, and bone age at diagnosis (negatively correlated). **Conclusions:** There was no significant difference in final height between treated and untreated girls. Triptorelin was effective in delaying the onset of menarche. Factors contributing to a better final height in treated girls were higher height at baseline, lower midparental height, and younger bone age.

## 1. Introduction

Adolescence is a transitional period during which physical and emotional changes occur, resulting in the development of the child into a mature person, capable of reproduction [1]. The term “puberty” refers to the physical changes associated with adolescence, resulting from the activation of the hypothalamic-pituitary-gonadal axis (HPG axis) and including the enlargement and activation of the gonads (ovaries, testes), the appearance of secondary sex characteristics and the acceleration of the growth rate [1]. In boys, the onset of puberty is marked by an increase in testicular size (>4 mL) and normally occurs between 9 and 14 years of age, with an average age of 11.5 years [1,2], whereas in girls, the onset of puberty is characterized by thelarche (Tanner Stage B2) and usually occurs at the age of 8–13 years with an average age of 10.5 years [1]. This activation of the hypothalamic-pituitary-gonadal axis is regulated by many factors, including genetics (50–80% correlation), environmental factors, socio-economic conditions, stress situations, obesity and chronic disease [1,3]. These factors can culminate in an earlier onset of puberty. Although central precocious puberty (CPP) remains the most common cause of PP, there are also cases of peripheral precocious puberty (PPP), where the HPG axis is not activated, and the clinical presentation results from exogenous exposure to androgens or estrogens, or from the endogenous autonomous production of sex hormones. “Precocious puberty” (PP) is used to describe the onset of thelarche (Tanner stage B2) in girls younger than 8 years of age or an increase in testicular volume ≥ 4 mL in boys younger than 9 years of age [4,5]. “Early puberty” (EP) occurs in girls at the age of 8–9 years and in boys at the age of 9–10 years, although this definition is not widely accepted [5,6]. PP and EP are much more common in girls than in boys and are among the most frequent reasons for visiting a Pediatric Endocrinology Outpatient Clinic. On consultation, families generally need to decide on the option of puberty suppression treatment with gonadotropin-releasing hormone analogues, such as triptorelin. Gonadotropin-releasing hormone analogues stabilize the progression of puberty and reduce both the growth rate and the rate of bone maturation bysuppressing the secretion of the natural hormone GnRH, and the activation of the hypothalamic-puitary-gonadal axis [7]. Their reported efficacy and satisfactory safety profile have made them the gold standard in the management of precocious puberty for many decades [5,7].

Precocious puberty may have several consequences, mainly psychological and behavioral, during the progression of adolescence [8]. It is also well established that early menarche, a frequent consequence of precocious puberty, is associated in later life with an increased risk of obesity, diabetes type 2, cardiovascular disease, breast and endometrial cancer [8].

For most parents, the main reported concern is a potentially shorter adult height, together with the psychological impact of early menarche. Scientific evidence shows that children with pubertal onset before the age of 6 clearly benefit from treatment in terms of height, but there are not many studies on the final height in children with precocious puberty after the age of 6 or with early puberty, which constitute the majority of cases in clinical practice [9,10].

Therefore, we retrospectively reviewed the impact of treatment with triptorelin on the final height of girls with precocious or early puberty, compared to untreated girls with the same conditions. Additionally, we explored factors that may contribute to the maximum effectiveness of the treatment in terms of final height.

## 2. Materials and Methods

This is a retrospective study based on medical records of girls who visited our Pediatric Endocrinology Outpatient Clinic with precocious or early puberty. Initial selection criteria included: (i) date of birth before 1 January 2011, (ii) thelarche Tanner stage 2 before the age of 9 years or menarche before the age of 11 years and (iii) laboratory confirmation of puberty (basal LH levels > 0.3 U/L or stimulated LH levels > 5 U/L after LHRH stimulation test). The latter was performed in a morning, fasting state by the administration of 100 µg of a GnRH analogue intravenously, and LH and FSH levels are measured at 0, 30, and 60 min. According to these criteria, 248 girls were found, and their guardians were contacted by phone to obtain extra information. Additional inclusion criteria for participation in further analysis were: (a) valid telephone number for contact, (b) parental and adolescent consent to participate in the study, (c) menarche 4 years ago or more reaching adult height, (d) recent objective height measurement (visit to our Outpatient Clinic and measurement of height or documented measurement by a health professional) and (e) absence of chronic disease or medication that may have affected final height. After applying the additional criteria, 178 females were eligible for the final statistical analysis. 34 of the initial 248 girls could not be contacted by phone, and for another 36 final height could not be accurately determined. These 178 females were divided into groups according to whether they received puberty suppression treatment with triptorelin (*n* = 85) or not (*n* = 93) and whether the onset of puberty was precocious (*n* = 85) or early (*n* = 93). Treatment with triptorelin was offered at a 3-month interval and age at treatment initiation and treatment duration was documented. For adolescents included in the final statistical analysis, height and weight during their outpatient visits were collected from their medical records, while BMI was calculated based on the formula BMI = weight (kg)/height^2^ (m). The midparental height was calculated based on the parents’ height measurements during the adolescent’s 1st visit and according to the formula: midparental height (for girls): ((father’s height − 13 cm) + mother’s height)/2 and midparental height (for boys): (father’s height + (mother’s height + 13 cm))/2. The absolute values of the anthropometric parameters were converted to standard deviation values (z-scores) based on Center for Disease Control reference values [11]. Bone age was estimated using a plain radiograph of the left hand, which was compared to reference radiographs from the Greulich and Pyle atlas [12].

### Statistical Analysis

The data were initially collected and recorded in a worksheet package and then statistically analyzed using Microsoft Excel for Mac, version 16.85 and IBM SPSS Statistics version 29.0.1.0 for Mac. These software packages were used for both statistical analysis and graphical visualization of the results.

The normality of the samples was assessed by the Shapiro-Wilk test for samples less than 50 and by the Kolmogorov-Smirnov test for samples greater than 50. For parameters with normal distribution, the Student’s *t*-test was used to compare the means of two different groups while for samples with skewed distribution, the Mann-Whitney test was used. The Chi-square test was used to compare the percentages. Linear correlations were tested by Pearson and Spearman correlations for parameters with normal and skewed distribution, respectively. Univariate and multivariate logistic regression analysis was performed to assess the effect of various parameters on the response to puberty suppression treatment. The limit of statistical significance was set to 0.05 (*p* < 0.05).

## 3. Results

The demographic and anthropometric data for the early puberty (*n* = 93) and precocious puberty groups (*n* = 85) are shown in Table 1. The mean standard deviation score (SDS) of BMI was statistically higher in the precocious puberty group than in the early puberty group (1.21 ± 0.56 kg/m^2^ vs. 0.90 ± 0.75 kg/m^2^, *p* = 0.006), as were the mean SDS of height and weight. Menarche occurred statistically earlier in the precocious puberty group compared to the early puberty group (10.68 ± 1.22 years vs. 11.12 ± 0.90 years, *p* = 0.005). No other statistically significant difference was observed between the two aforementioned groups.

Additionally, the data for the study population divided between untreated (*n* = 93) and treated (*n* = 85) girls is set out in Table 2. For the treated girls, mean age at treatment initiation was 8.42 ± 0.75 years and mean treatment duration was 1.83 ± 0.72 years. The mean SDS of midparental height of treated girls was statistically lower compared to the mean standard deviation of midparental height of untreated girls (−0.20 ± 0.81 vs. +0.03 ± 0.73, *p* = 0.050). Height, weight and BMI were in absolute values higher for girls not receiving triptorelin than for girls who received treatment. When standard deviations (SDS) of anthropometric parameters were examined, only height remained statistically higher in untreated girls compared to treated girls (1.29 ± 0.99 vs. 0.77 ± 1.01, *p* < 0.001). Final height, as well as the difference in SDS between final and midparental height, did not differ significantly between the two groups.

The data for the study population who experienced early puberty (*n* = 93) was finally classified into two subgroups according to whether they were (*n* = 43) or were not treated with triptorelin (*n* = 50): see Table 3.

The age of menarche was significantly earlier in untreated girls (10.69 ± 0.75 years vs. 11.61 ± 0.82 years, *p* < 0.001). Untreated girls with early puberty had statistically higher height and weight at baseline compared to treated girls, as reflected in both absolute values and SDS. Finally, there was no statistically significant difference in final height or difference from midparental height in girls with early puberty, whether they received treatment or not.

Similarly, we sorted data for the study population that experienced precocious puberty into two subgroups according to whether they were treated with triptorelin (*n* = 42) or not—see Table 4.

The age of menarche was significantly earlier in untreated girls with precocious puberty (9.86 ± 0.86 years vs. 11.53 ± 0.90 years, *p* < 0.001). Midparental height of untreated girls was statistically higher compared to midparental height of treated girls in both absolute values (164.88 ± 4.59 cm vs. 161.87 ± 4.19 cm, *p* = 0.003) and SDS. Untreated girls had statistically higher height at baseline compared to treated girls, as reflected in both absolute values and SDS. Finally, there was no statistically significant difference in final height or in the difference from the midparental height in girls with precocious puberty, whether or not they received triptorelin.

In order to determine the factors that may influence the effectiveness of puberty suppression treatment in terms of final height, the difference of the standard deviation of the final height from the midparental height (ΔHeight (Final − Midparental height) SDS was chosen as the dependent variable, and it was considered that the higher the value of this parameter, the better the outcome in terms of final height. The linear correlations of the various parameters that may independently influence this parameter (univariate analysis) are shown in the Table 5.

**Figure 1:**
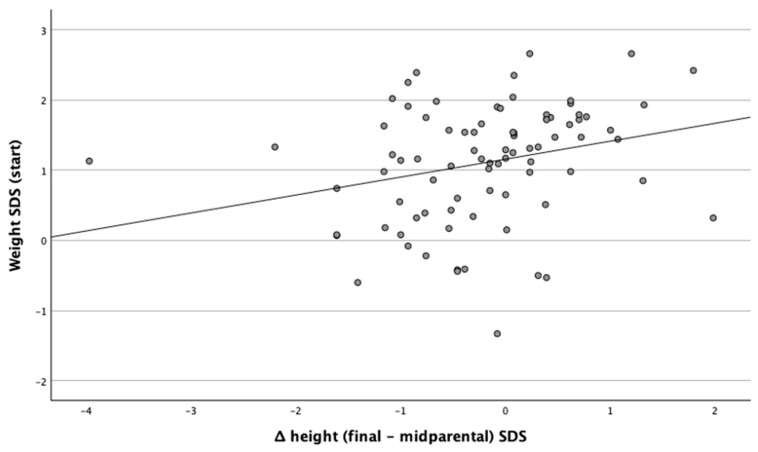
Positive linear correlation between Weight SDS at the initiation of treatment and the difference of final − midparental height SDS (*r* = 0.345, *p* = 0.001).

The standard deviation of weight at the start of treatment was positively related to the ΔHeight (Final − Midparental) SDS, Figure 1.

The SDS of midparental height was marginally significantly related, and related negatively, with ΔHeight (Final − Midparental) SDS. The multivariate study to predict the highest ΔHeight (Final − Midparental) SDS showed that the standard deviation of height at the start of treatment, standard deviation of midparental height and bone age are the three most important parameters, predicting 46.9% of the variability of the variable, Table 6. Higher height at baseline, lower midparental height and younger bone age best predict the greatest difference in the ΔHeight (Final − Midparental) SDS.

## 4. Discussion

Girls with precocious or early puberty represent a large number of cases presented to the pediatric endocrinology outpatient clinic, due to parental concerns about both their final height and their psychosocial maturation. GnRH analogues, such as triptorelin, are an established treatment that helps delay the progression of puberty with proven benefit for girls who experience precocious puberty before the age of 6 years. However, there is insufficient evidence in the literature concerning girls with onset of puberty between 6–8 years of age or early puberty, which are the most common cases in clinical practice [9,10]. The present study aimed to illustrate the impact of triptorelin on the final height of girls with precocious or early puberty, compared with a group of girls with precocious or early puberty who did not receive puberty suppression treatment.

In our study, the diagnosis of precocious or early puberty in many girls was based on a peak LH level exceeding 5 IU/L following intravenous GnRH administration, measured at 30 and/or 60 min. Although alternative cut-off values—such as 4.1 IU/L—have also demonstrated high sensitivity and specificity, numerous studies consistently support a stimulated LH threshold of ≥5 IU/L as diagnostic for central precocious puberty [13,14].

Regarding the primary endpoint of our study, final height and SDS of the difference between final and midparental height (ΔHeight (final − midparental) SDS) did not differ significantly between treated and untreated girls. Results were similar when we studied precocious and early puberty subgroups separately. These findings are consistent with the results of the meta-analysis by Bertelloni et al., which included 8 studies of final height in a total of 483 girls with early or precocious puberty (ages 7–10 years), either treated or not treated with GnRHas (triptorelin or leuprolide), where there was no significant benefit from treatment in terms of the final height compared to the control groups [15]. However, in a more recent meta-analysis, there minwas a significantly improved adult height in girls with early and precocious puberty (6.3–9 years old), who were treated with GnRHas [16]. In another retrospective study from China, girls with precocious or early puberty treated with leuprolide, showed improvement in final height and delay of bone maturation, although this was a study without a control group [17]. In addition, a study of 42 patients (33 girls and 9 boys) with central precocious puberty from Korea showed an improvement in predicted final height in the first 18 months of treatment with a triptorelin depot of 22.5 mg every six months [18]. The main limitation of this study was that study population did not reach the final height, so the true effect of treatment on growth could not be fully assessed.

It is becoming clear that it is methodologically difficult to assess the true effect of GnRHas on the final height of children with early or precocious puberty. First, there is great heterogeneity in the characteristics of patients undergoing puberty suppression therapy, such as chronological and bone age at the start of treatment, duration of treatment, rate of progression of puberty and their anthropometric characteristics. In addition, different methods are used to assess final height, such as comparison with predicted height or midparental height or the height of a control group. Nevertheless, the calculation of the predicted height, based on the Bayley-Pinneau method, tends to overestimate the final height of patients with precocious puberty who did not receive treatment [10]. Finally, most published studies are retrospective observational studies and not randomized controlled trials. This implies that the decision for treatment was based on the wishes of patients and their parents, without specific criteria for initiation of treatment or a specific protocol, and with the risk of non-compliance or early discontinuation.

Despite these factors, we wanted to see if there were common clinical features in girls with precocious and early puberty where their families chose to proceed with treatment. We found that the mean standard deviation of midparental height of treated girls was statistically lower compared to the mean standard deviation of the midparental height of untreated girls, while the mean standard deviation of height was statistically higher in untreated girls compared with the treated girls. This finding suggests that the parents who chose treatment were the shortest and also had the shortest children, factors that may have influenced their decision.

In addition to final stature, another important factor to consider when deciding to initiate GnRHas suppression therapy is the psychological effect of an earlier physical maturation of the child. As expected, the age of menarche was statistically younger in the group of untreated girls compared to the group that received triptorelin in our study, indicating the success of the treatment in delaying physical maturation. Adolescents are characterized by a desire to be accepted by their peers and not to be different from them. A review article by Graber et al. points out that girls who mature physically earlier than their peers are at increased risk of developing psychological problems, including depression, of illicit substance use and of earlier sexual activity [19]. Furthermore, a study by Karen Rudolph et al. at the University of Illinois, who followed more than 160 teenagers, boys and girls, for three years, found increased rates of depressive symptoms in girls with precocious or early puberty compared to their peers [20].

Concerning the factors contributing to better outcomes regarding final height, we found that higher height and younger bone age at the start of treatment, as well as shorter midparental height, better predict the greater difference in SDS between final and midparental height, thus better treatment efficacy. Other studies have also attempted to highlight the factors influencing the effectiveness of GnRHas treatment in children with precocious puberty. Similarly with our findings, Carel et al., evaluated baseline height and midparental height as parameters influencing treatment outcome, as well as bone age at the end of treatment [21]. Klein et al., using final height in cm as an indicator of treatment efficacy, positively correlated higher predicted height, longer duration of treatment and higher annual growth rate in the last year of treatment, while advanced bone age at the start of treatment and late initiation of treatment were negatively associated with the efficacy of GnRHas [22]. Park et al. reported in their meta-analysis duration of treatment as the most important determinant [16], whereas a recent retrospective study in Taiwan found that younger age at the initiation of treatment and faster growth velocity during treatment were associated with better height gain [23]. Finally, Trujillo et al. have suggested individualized monitoring during GnRHa treatment, based on the changes of predicted adult height [24]. To sum up, most studies highlight the importance of early diagnosis and initiation of treatment in order to achieve optimal results in terms of final height.

Our study has certain limitations. First, it is a retrospective, observational study, involving patients from a single center, and not a randomized clinical trial with a specific protocol, which would ensure the homogeneity of patients’ characteristics at baseline and the treatment process. That means that the decision for treatment was based on the willingness of the children’s caregivers, as well as on compliance, possibly also on the time of discontinuation of treatment. Another limitation is that the final height was in some cases measured by health professionals, but not in our Pediatric Endocrinology Outpatient Clinic. Moreover, certain forms of precocious puberty are known to have an underlying genetic etiology, which was not investigated in this study. The absence of genetic testing may represent a limitation, as such factors could potentially influence not only pubertal timing but also parental height characteristics. Finally, we did not examine factors related to the patients’ lifestyles, such as physical activity and dietary habits.

Despite the limitations, to our knowledge, this is the first study evaluating the effect of triptorelin on the final height of a considerable number of girls with early or precocious puberty in the Greek population, as well as the factors that positively influence the outcome in terms of final height, using as a criterion the standard deviation difference of the final height from the midparental height.

## 5. Conclusions

Early/precocious puberty in girls is one of the most common reasons for visiting the Pediatric Endocrinology Outpatient Clinic. GnRH analogues are the appropriate treatment, as they delay the progress of puberty, with a satisfactory safety profile [5,25,26]. The main goal when deciding on treatment is to prevent the psychological effects of premature physical maturation of children. However, the final height of these children is a common concern among their parents. In our study, there was no statistically significant difference in final height between girls who received triptorelin and those who did not. On the other hand, there was a statistically significant difference in the age of menarche, which was younger in untreated girls compared to treated girls. This finding confirms the success of the treatment in delaying physical maturation.

Factors found to contribute to better treatment outcome regarding final height were higher height at diagnosis, shorter midparental height, and younger bone age, and should be considered when making an individualized decision to treat or not to treat. More targeted studies on the impact of GnRHas on the final height of children—girls and boys—with early and precocious puberty, as well as the factors influencing it, will contribute to a better-informed decision by parents.

## Figures and Tables

**Table 1:** Anthropometric and Demographic data of girls classified in precocious puberty and early puberty group (statistically significant differences are shown in bold).

Parameter	Early Puberty	Precocious Puberty	*p*
N	93	85	
Midparental height (cm)	162.29 ± 5.31	163.43 ± 4.62	0.139
Midparental height SDS	−0.16 ± 0.82	−0.01 ± 0.72	0.145
Adoption *n* (%)	3 (3.23)	6 (7.06)	0.240
Age of menarche (years)	11.12 ± 0.90	10.68 ± 1.22	**0.005**
Final height (cm)	161.06 ± 6.74	161.52 ± 6.32	0.681
Final height SDS	−0.35 ± 1.04	−0.28 ± 0.97	0.681
ΔHeight SDS (Final − Midparental)	−0.19 ± 0.78	−0.29 ± 0.94	0.448
Height at diagnosis (cm)	139.92 ± 8.63	138.51 ± 10.92	0.339
Height at diagnosis SDS	0.75 ± 0.99	1.36 ± 0.98	**<0.001**
Weight at diagnosis (kg)	39.00 ± 9.68	38.58 ± 9.57	0.814
Weight at diagnosis SDS	1.00 ± 0.85	1.50 ± 0.62	**<0.001**
BMI at diagnosis (kg/m^2^)	19.62 ± 3.00	19.81 ± 2.65	0.549
ΒΜΙ at diagnosis SDS	0.90 ± 0.75	1.21 ± 0.56	**0.006**

**Table 2:** Anthropometric and Demographic data of girls classified in non-treatment and treatment group (statistically significant differences are shown in bold).

Parameter	Non-Treatment	Treatment	*p*
N	93	85	
Midparental height (cm)	163.52 ± 4.77	162.05 ± 5.21	0.058
Midparental height SDS	0.03 ± 0.73	−0.20 ± 0.81	**0.050**
Adoption *n* (%)	4/93 (4.30%)	5/85 (5.88%)	0.630
Age of menarche (years)	10.31 ± 0.91	11.57 ± 0.86	**<0.001**
Final height (cm)	161.74 ± 6.25	160.77 ± 6.83	0.453
Final height SDS	−0.24 ± 0.96	−0.39 ± 1.05	0.453
ΔHeight SDS (Final − Midparental)	−0.28 ± 0.83	−0.20 ± 0.89	0.243
Height at diagnosis (cm)	144.19 ± 9.33	133.88 ± 7.13	**<0.001**
Height at diagnosis SDS	1.29 ± 0.99	0.77 ± 1.01	**<0.001**
Weight at diagnosis (kg)	43.01 ± 10.09	34.13 ± 6.36	**<0.001**
Weight at diagnosis SDS	1.36 ± 0.73	1.11 ± 0.84	0.077
BMI at diagnosis (kg/m^2^)	20.39 ± 2.97	18.96 ± 2.48	**<0.001**
ΒΜΙ at diagnosis SDS	1.11 ± 0.61	0.99 ± 0.75	0.689

**Table 3:** Anthropometric and Demographic data of girls with early puberty classified in non-treatment and treatment group (statistically significant differences are shown in bold).

Parameter	Non-Treatment	Treatment	*p*
N	50	43	
Midparental height (cm)	162.35 ± 4.66	162.21 ± 6.04	0.899
Midparental height SDS	−0.15 ± 0.72	−0.17 ± 0.93	0.911
Adoption *n* (%)	2/50 (4.00%)	1/43 (2.33%)	0.649
Age of menarche (years)	10.69 ± 0.75	11.61 ± 0.82	**<0.001**
Final height (cm)	161.25 ± 7.24	160.84 ± 6.19	0.768
Final height SDS	−0.32 ± 1.11	−0.39 ± 0.96	0.757
ΔHeight SDS (Final − Midparental)	−0.17 ± 0.83	−0.22 ± 0.71	0.778
Height at diagnosis (cm)	143.85 ± 8.50	135.44 ± 6.34	**<0.001**
Height at diagnosis SDS	0.95 ± 0.99	0.52 ± 0.96	**0.038**
Weight at diagnosis (kg)	42.73 ± 10.06	34.56 ± 7.08	**<0.001**
Weight at diagnosis SDS	1.16 ± 0.74	0.81 ± 0.94	**0.048**
BMI at diagnosis (kg/m^2^)	20.34 ± 2.95	18.76 ± 2.87	**0.011**
ΒΜΙ at diagnosis SDS	1.02 ± 0.63	0.76 ± 0.86	0.088

**Table 4:** Anthropometric and Demographic data of girls with precocious puberty classified in non-treatment and treatment group (statistically significant differences are shown in bold).

Parameter	Non-Treatment	Treatment	*p*
N	43	42	
Midparental height (cm)	164.88 ± 4.59	161.87 ± 4.19	**0.003**
Midparental height SDS	0.25 ± 0.70	−0.24 ± 0.66	**0.002**
Adoption *n* (%)	2/43 (4.65%)	4/42 (9.52%)	0.381
Age of menarche (years)	9.86 ± 0.86	11.53 ± 0.90	**<0.001**
Final height (cm)	162.30 ± 4.87	160.71 ± 7.51	0.418
Final height SDS	−0.16 ± 0.75	−0.40 ± 1.15	0.418
ΔHeight SDS (Final − Midparental)	−0.39 ± 0.83	−0.19 ± 1.04	0.315
Height at diagnosis (cm)	144.59 ± 20.38	132.28 ± 7.61	**<0.001**
Height at diagnosis SDS	1.68 ± 0.85	1.05 ± 1.01	**0.002**
Weight at diagnosis (kg)	43.35 ± 10.24	33.70 ± 5.65	**<0.001**
Weight at diagnosis SDS	1.59 ± 0.65	1.41 ± 0.59	0.189
BMI at diagnosis (kg/m^2^)	20.44 ± 3.02	19.16 ± 2.04	**0.025**
ΒΜΙ at diagnosis SDS	1.20 ± 0.58	1.22 ± 0.55	0.880

**Table 5:** Univariate linear associations of various parameters likely to influence treatment efficacy on final height with the ΔSDS (Final − Midparental height).

Parameter	R	*p*
Midparental height	−0.216	0.055
Age at initiation of treatment	−0.048	0.660
Age at the end of treatment	−0.151	0.170
Duration of treatment	−0.067	0.545
Height at initiation SDS	0.177	0.104
Weight at initiation SDS	0.345	0.001
BMI at initiation SDS	0.187	0.088
Bone age at initiation	0.069	0.542

**Table 6:** Multivariate analysis of various parameters likely to influence treatment efficacy on final height with the ΔSDS (Final − Midparental height).

Parameter	Unadjusted β	*p*
Height SDS (initiation)	0.660	<0.001
Midparental height (SDS)	−0.107	<0.001
Bone age (initiation)	−0.166	0.037

## Data Availability

The data that support the current study are available upon reasonable request.

## Abbreviations

The following abbreviations are used in this manuscript:

PP	Precocious Puberty
EP	Early Puberty
SD	Standard Deviation
GnRHa	Gonadotropin Releasing Hormone analogues
